# Management of cryptococcal meningitis in HIV-infected patients: Experience from western India

**DOI:** 10.4103/2589-0557.68996

**Published:** 2010

**Authors:** Atul K. Patel, Ketan K. Patel, Rajiv Ranjan, Shalin Shah, Jagdish K. Patel

**Affiliations:** Infectious Diseases Consultant, Infectious Diseases Clinic, Adit Molecular Diagnostics, “Vedanta” Institute of Medical Sciences, Navarangpura, Ahmedabad - 380 009, India; Adit Molecular Diagnostics, “Vedanta” Institute of Medical Sciences, Navarangpura, Ahmedabad - 380 009, India; 2 Neurology Department, Sterling Hospital, Memnagar, Ahmedabad - 380 052, India

**Keywords:** Amphotericin B, cryptococcal meningitis, HIV

## Abstract

**Introduction:**

Cryptococcal meningitis is one of the acquired immunodeficiency syndrome defining infections with high mortality. Amphotericin B is the preferred drug for induction therapy. Despite advances in human immunodeficiency virus (HIV) treatment, Antiretroviral Treatment (ART) roll-out programs and availability of amphotericin B, cryptococcal meningitis remains an important cause of mortality in the African and other developing countries.

**Materials and Methods:**

We carried out a prospective observational study to determine the treatment response rate, tolerability and outcome of patients with cryptococcal meningitis in HIV treated with amphotericin B. Descriptive statistic was used to analyze the data.

**Results:**

A total of 27 patients were diagnosed with cryptococcal meningitis during the study period. Headache (96.29%) was the single most common presenting symptom of cryptococcal meningitis in HIV-infected patients, followed by vomiting (77.77%) and fever (66.66%). Cerebrospinal fluid (CSF) routine and microscopic examination was within normal limits in six patients. CSF became sterile on the 12th day of Amphotericin B in 55.55% of the patients while 33.33% had positive CSF cultures. Patients were started with ART after achieving sterile CSF and tolerated at least 2 weeks of fluconazole consolidation treatment and were free from symptoms. Median time for antiretroviral treatment initiation was 35 (14–90) days after completion of Amphotericin B treatment. One patient developed immune reconstitution inflammatory syndrome (IRIS) after ART.

**Conclusions:**

We found that the recommended 2 weeks induction treatment with Amphotericin B monotherapy for HIV patients with cryptococcal meningitis in resource-limited settings may be suboptimal for at least one-third of the patients. Extending the therapy to 3 weeks is likely to result in sterilization of the CSF in a majority of these patients. This finding requires confirmation by a larger sample size in appropriately powered studies. Delaying ART initiation by at least 2 weeks after amphotericin B treatment may decrease the incidence of IRIS.

## INTRODUCTION

Cryptococcal meningitis is one of the acquired immunodeficiency syndrome-defining infections. Although opportunistic infections are declining after the availability of antiretroviral treatment in developing countries, many patients are diagnosed with cryptococcal meningitis due to late presentation.[1]

Cryptococcus is a ubiquitous environmental fungus in many parts of the world. This opportunistic fungal infection usually produces infection in immunocompromised hosts like human immunodeficiency virus (HIV) subjects, diabetics, solid organ transplant recipients and patients receiving immunosuppressive treatment. Headache may be the only symptom as presenting feature of cryptococcal meningitis in HIV. It is crucial to understand that, in HIV-infected patients, cryptococcal meningitis can be present even in the absence of fever and meningismus. More than 75% of the patients with cryptococcal meningitis in HIV-infected patients have fever, most also have headache, but a substantial number do not have these manifestations. Some patients may present with isolated obtundation, cranial neuropathies, cognitive dysfunction or seizures.[2,3] Cryptococcal meningitis can have a very indolent, subacute presentation in HIV-infected patients, requiring a high index of suspicion. Diagnosis is performed by cerebrospinal fluid (CSF) examination with India Ink preparation, cryptococcal antigen test (CrAg) in CSF/serum and CSF culture. Neuroimaging also helps in diagnosing associated complications of cryptococcal meningitis, like cryptococcomas, infarcts, hydrocephalus, etc. Amphotericin B is the drug of choice as induction therapy. Despite advances in HIV treatment, ART roll-out programs and availability of Amphotericin B, cryptococcal meningitis remains an important cause of mortality in the African and other developing countries.[4–6]

## MATERIALS AND METHODS

We carried out a prospective observational study to determine the treatment response rate, tolerability and outcome of patients with cryptococcal meningitis in HIV patients treated with amphotericin B. HIV patients attending the clinic between December 2006 and January 2010 and diagnosed with cryptococcal meningitis were prospectively followed-up, especially with regard to clinical presentation, CD4 count, CSF abnormalities, response to treatment, time to start ART and any IRIS. Data were recorded in Microsoft Excel sheets by the treating physicians at the Infectious Diseases Clinic.

### Diagnosis

Suspected patients underwent CSF examination, India ink preparation, CSF CrAg and CSF culture. Patients with CSF cryptococcal antigen positivity with or without India Ink positivity were treated. Patients presenting in altered sensorium with a focal neurological deficit or intense headache not improving despite a lumber tap were subjected to neuroimaging.

### Treatment

All patients were hospitalized and a central line was placed. Conventional Amphotericin B 0.7 mg/kg/day (Amphotericin B deoxycholate) was used as induction therapy. Amphotericin B was infused in 5% dextrose over 8 h and the patients received 500 ml of 0.9% Normal saline (NS) before infusion to reduce Amphotericin B-related toxicity. In addition to this, patients were encouraged to ensure liberal intake of fluids and coconut water, citrus fruits and banana daily. Amphotericin toxicity was monitored with twice-weekly creatinine and K level. Patients with hypokalemia and requiring K supplementation were tested daily. These patients’ calcium and magnesium levels were also checked and corrected if required. Amphotricin B infusion was discontinued in patients with rising creatinine levels and resumed once creatinine levels touched baseline. Fever and chills related to Amphotericin B infusion were treated with inj hydrocortisone 50 mg IV sos. Raised intracranial pressure (ICP) was controlled with lumber tap and mannitol. Frequency of lumber tap was guided by patient’s clinical symptoms. Fifteen to 20 ml of CSF was removed at one time to control the raised ICP.

### Successful response

Treatment response was defined as sterile CSF culture at day 12. Patients with persisting positive CSF cultures were continued on Amphotericin B infusion for one more week and evaluated with repeat CSF culture. They were then consolidated with 400 mg of fluconazole for 8 weeks and maintained on fluconazole 200 mg/day.

Patients were followed-up for a minimum of 3 months after diagnosis of cryptococcal meningitis. Descriptive statistics were used to analyze the data.

## RESULTS

A total of 27 patients were diagnosed with cryptococcal meningitis during the study period. Baseline characteristics of these patients are described in [Table 1].

**Table 1:** Baseline characteristics of the patients

Parameters	Results (n = 27)
Age in years, median (range)	37 (26–45)
Sex:	
Male	23 (85.19%)
Female	4 (14.81%)
Weight (kg)	50 (30–70)
Serum creatinine	0.82 (0.82–1.53)
CD4 count	73 (8–193)
Symptoms:	
Headache	26 (96.29%)
Seizure	9 (33.33%)
Altered sensorium	9 (33.33%)
Focal neurological deficit (FND)	4 (14.81%)
Fever	18 (66.66%)
Vomiting	21 (77.77%)
Dizziness	10 (37.03%)
Associated OIs:	
TB	6 (22.22%)
Candida	14 (51.85%)
Diarrhea	3 (11.11%)
PCP	1 (3.70%)
CSF abnormalities	
Protein	90 (26–360)
Sugar	44 (13–83)
Cells	5 (1–180)
India Ink	26 (96.29%)
Cryptococcal antigen titer	8 (2–16)
Culture at baseline	27 (100%)

Headache (96.29%) was the single most common presenting symptom of cryptococcal meningitis in HIV-infected patients, followed by vomiting (77.77%) and fever (66.66%). Cryptococcal meningitis was the first presenting illness in 22 (78.57%) patients, while six (21.43%) patients developed cryptococcal meningitis on ART. Of these, three had unmasking of cryptococcal meningitis and three patients defaulted ART with clinical failure. Two patients had two episodes of cryptococcal meningitis, having defaulted ART and responded both times to the same regimen.

CSF routine and microscopic examination was within normal limits in six patients, while 20 had elevated proteins, six patients’ CSF sugar was <50% of blood sugar and the CSF white blood cell (WBC) count was >5 in 13 patients.

Magnetic resonance imaging (MRI) of the brain was performed in 11 patients. MRI abnormalities included cerebral atrophy, left parietal irregular hyperintense lesion, cerebritis with meningeal inflammation, white matter lesion, cryptococcoma and cranial venous sinus thrombosis in one patient each, while it was normal in five patients.

Adverse drug reactions to Amphotericin B were generally mild and patients tolerated the drug well. Six patients developed renal dysfunction (defined as any degree of elevation of serum creatinine), which improved after drug discontinuation. These patients safely completed the prescribed course of Amphotericin B. Therapeutic lumber puncture was highly effective in taking care of severe headache. Eleven patients required >2 therapeutic lumber taps for their headache (range, 2–7 lumber taps).

CSF became sterile on the 12^th^ day of Amphotericin B in 55.55% of the patients while 33.33% had positive CSF cultures and were treated with an extended course of Amphotericin B by one more week. All these patients’ CSF became sterile on day 19. Two patients’ CSF grew cryptococcus after 1 week of incubation and were continued on fluconazole consolidation regimen. Three patients (11.11%) were lost to follow-up (due to financial constraints, they opted for treatment at their native place and did not present for follow-up) and one patient (cryptococcoma and meningoencephalitis) died in the hospital during treatment.

### Art

Patients were started with ART after achieving sterile CSF and tolerated at least 2 weeks of fluconazole consolidation treatment and were free from symptoms. Median time for antiretroviral treatment initiation was 35 (14–90) days after completion of Amphotericin B treatment. One patient developed IRIS after ART. Symptoms of IRIS were fever, headache and lymph node TB IRIS.

## DISCUSSION

Cryptococcal disease remains an important presenting illness in HIV-infected patients in India. We describe a prospective series of cryptococcal meningitis in HIV patients at a tertiary referral center in Gujarat. Headache, vomiting and fever were three important presenting symptoms of cryptococcal meningitis in our series. CSF findings in our series, in contrast to the Kumar *et al*. study, were normal in six (21.43%) subjects, whereas they showed elevated proteins in 71.43% vs. 45% in the Kumar *et al*. study, CSF sugar was low in 21.43% vs. 75% in the Kumar *et al*. study and CSF WBC count was >5 in 13 (46.43%) patients with 100% lymphocytic predominance as against 55% in Kumar’s study. CSF India Ink examination was positive in 96.29% vs. 85% in the Kumar *et al*. study while CrAg was positive in all patients in both studies. We had a 100% culture positivity vs. 90% in the Kumar *et al*. study.

Treatment with flucytosine plus Amphotericin B is recommended in many reference books and guidelines.[7,8] The addition of flucytosine results in faster sterilization of the CSF and fewer relapses than with the use of Amphotericin B alone, but there is no difference in mortality at the 14^th^ day between the two regimes.[7,9] However, for patients receiving antiretroviral therapy, the relapse-reduction advantage from adding flucytosine is likely to be very small.

In the present series, Amphotericin B monotherapy was effective in sterilizing CSF at the 12th day in 55.55% of the patients; one patient received 3^rd^ week of Amphotericin B with 5 flucytosine 100 mg/kg/day while two (7.40%) patients had scanty growth at day 5 and had been started on fluconazole consolidation and were continued the same till they were asymptomatic. Six (22.22%) patients received 3 weeks of Amphotericin B to sterilize CSF. All patients received fluconazole 400 mg for 8 weeks as consolidation followed by 200 mg daily as maintenance therapy.

CSF sterilization is the goal of therapy for cryptococcal infection. However, it is common for HIV-infected patients to have positive cultures at the end of induction therapy. In one study, 40% of the patients completing induction therapy with Amphotericin B plus flucytosine and 28% of those completing consolidation therapy with fluconazole had positive CSF cultures.[10] In our series, 55.55% of the patients achieved sterile CSF at the 12^th^ day of Amphotericin B monotherapy. 25.93% patients required 3 weeks of Amphotericin B treatment to achieve sterile CSF. This finding suggests that patients should be subjected for CSF culture before discontinuing Amphotericin B as induction therapy. Persistent cryptococcal infection might lead to IRIS following ART initiation. Untreated cryptococcal meningitis is universally fatal. Studies from African countries have reported a very high mortality, while the morbidity and mortality in HIV patients with cryptococcal meningitis in developed countries is 2.5–15%.[10–13] Common explanation given in various studies include late presentation with low CD4 counts, Amphotericin B toxicity, uncontrolled raised ICP, focal neurologic deficit and IRIS. A high mortality rate of 33.87% at week 4 was observed in Kwa Zulu Natal by Lightowler.[14] In another study, the mortality was 14% at 2 weeks and 22% at 10 weeks.[15] A study from Thailand showed a 16% mortality at 2 weeks and 24% at 4 weeks.[16] While the Kumar *et al*. study from north India reported a 7.5% mortality, in the present series, it was 3.57%, which compares well with the series reported from the developed countries.[17] An established aggressive format of management of patients with cryptococcal meningitis at our tertiary care center under expert supervision, which includes a central line placement, adequate pre-hydration and prolonged Amphotericin B infusion to reduce drug-related toxicity, avoiding concomitant nephrotoxic drugs, control of raised ICP by therapeutic lumber tap as and when required and reducing the chances of IRIS by delaying the commencement of ART by 2 weeks after achievement of a sterile CSF, probably resulted in reduced mortality in our study as compared to that of other studies. It has been well studied that continuous slow infusion of Amphotericin B and pre-hydration is associated with lesser toxicity and a better outcome.[18]

The rehospitalization rate is also high in some of the studies. We had to readmit three (11.11%) patients, two with neurological deterioration and one with hypokalemia. The reported incidence of IRIS in patients treated for cryptococcal meningitis varies by geographic region, and ranges between 4.2 cases and 18.2 cases per 100 person-years of follow-up.[19–21] The results of a recent prospective study showed a higher incidence of cryptococcus-associated IRIS at 47 cases per 100 person-years of follow-up (95% confidence interval [CI]: 25–80).[22] The variability in incidence is related, to some degree, to the differences in the definitions of IRIS, the burden of disease and the intensity of follow-up. Studies from developing countries favor a delay of 10 weeks after initiation of treatment for starting ART.[23,24] In our series, the incidence of IRIS was low (3.7%), probably due to the delay in initiation of ART by at least after 2 weeks after attainment of a sterile CSF.

## CONCLUSIONS

We found that the recommended 2-week induction treatment with Amphotericin B monotherapy for HIV patients with cryptococcal meningitis in resource-limited settings may be suboptimal for at least one-third of the patients. Extending the therapy to 3 weeks is likely to result in sterilization of the CSF in a majority of these patients. This finding requires confirmation by a larger sample size in appropriately powered studies. Delaying ART initiation by at least 2 weeks after Amphotericin B treatment may decrease the incidence of IRIS.
